# Evaluating early cardiology involvement and survival outcomes across NT‐proBNP levels: An island‐wide retrospective cohort study

**DOI:** 10.1002/ejhf.70060

**Published:** 2025-10-09

**Authors:** Chris Brown, Brian Wang, Carolina Almeida, Tim Sims, Marco Vidal, Pierre Le Page, Oliver J. Rider, Andrew R.J. Mitchell, John A. Henry

**Affiliations:** ^1^ Department of Cardiology Jersey General Hospital St. Helier Jersey; ^2^ Laboratory Services Jersey General Hospital St. Helier Jersey; ^3^ Oxford Centre for Clinical Magnetic Resonance Research, Division of Cardiovascular Medicine, Radcliffe Department of Medicine University of Oxford Oxford UK

Heart failure (HF) remains a significant global health burden, affecting over 64 million individuals and is associated with high morbidity, mortality, and healthcare costs.^1^ Despite improvements in medical therapy, prognosis continues to be poor, with 5‐year mortality rates around 50%.^2^ Delayed diagnosis and intervention contribute to these poor outcomes, emphasizing the importance of early identification and specialist involvement. N‐terminal pro‐B‐type natriuretic peptide (NT‐proBNP) measurement is well‐established for diagnosis and risk stratification in suspected or established HF,^3^ and current guidelines recommend specialist cardiology referral for patients with elevated levels.^4^ However, evidence quantifying the impact of timely cardiology review on survival is limited.

This retrospective cohort study analysed all NT‐proBNP tests performed on the island of Jersey between test introduction in January 2013 and December 2024, linking results to mortality data. All adult patients with at least one NT‐proBNP measurement were identified from laboratory records. For patients with multiple tests, the earliest was considered the index measurement. We excluded patients with end‐stage renal failure requiring dialysis and those with congenital heart disease. The proportion of the population at risk of HF, defined as those with at least one of hypertension, obesity, atrial fibrillation, coronary heart disease, chronic kidney disease or diabetes was calculated from Public Health reports.^5^ The study received favourable ethical approval (2023/HCSREC/10).

The NT‐proBNP values were categorized: <400, 400–2000, 2000–5000, and >5000 pg/ml. One‐year mortality was analysed using a multivariable time‐dependent Cox proportional hazards model. This model included early cardiology involvement (defined as outpatient review within 28 days of index NT‐proBNP test) as a time‐dependent covariate, and also adjusted for age, sex, and baseline NT‐proBNP category; the primary results from this model are presented in *Figure* *1*. To further assess the impact of early cardiology involvement and minimize immortal time bias, propensity score matching and landmark analysis were also employed. All analyses used R version 4.4.2, with *p* < 0.05 considered statistically significant.

**Figure 1:**
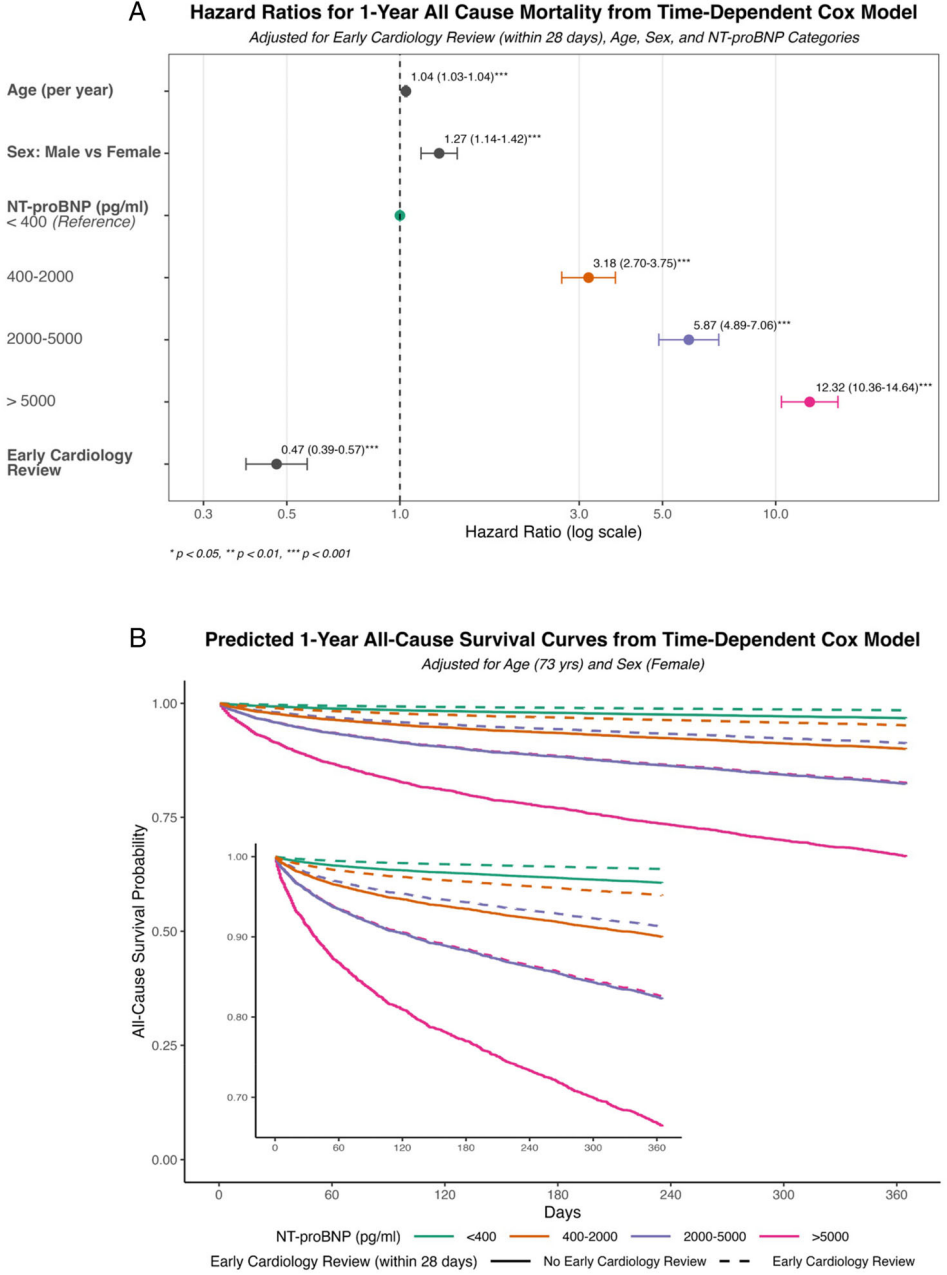
(*A*) Hazard ratios for 1‐year all‐cause mortality from time‐dependent Cox model, adjusted for early cardiology review (within 28 days), age, sex and N‐terminal pro‐B‐type natriuretic peptide (NT‐proBNP). (*B*) Predicted 1‐year all‐cause survival curves from time‐dependent Cox model adjusted for age and sex.

We identified 14 333 patients with NT‐proBNP measurements; 14 231 were included after exclusions. The proportion of the population deemed to be at risk of HF varied annually between 22% and 27%, with the percentage of the population undergoing NT‐proBNP increasing from 0.1% at test introduction in 2013 to 7.9% in 2024. Distribution across NT‐proBNP categories was: 64.2% <400 pg/ml, 21.3% 400–2000 pg/ml, 8.0% 2000–5000 pg/ml, and 6.5% >5000 pg/ml. Patients with higher NT‐proBNP levels were older (mean age 65.9 years [<400 pg/ml] vs. 80.6 years [>5000 pg/ml]) and more likely male (45.1% [<400 pg/ml] vs. 56.7% [>5000 pg/ml]).

The results from the multivariable time‐dependent Cox model, which adjusted for age, sex, NT‐proBNP category, and early cardiology review (with the latter as a time‐dependent covariate), are presented in *Figure* *1*. This model showed that NT‐proBNP category was a powerful independent predictor of 1‐year mortality. Compared to <400 pg/ml, adjusted hazard ratios (aHRs) from this model were 3.18 (95% confidence interval [CI] 2.70–3.75) for 400–2000 pg/ml, 5.87 (4.89–7.06) for 2000–5000 pg/ml, and 12.32 (10.36–14.64) for >5000 pg/ml. One‐year mortality was 3.6% (95% CI 3.2–4.1) for <400 pg/ml, 14.6% (13.3–16.0) for 400–2000 pg/ml, 24.9% (22.3–27.7) for 2000–5000 pg/ml, and 44.2% (40.8–47.6) for >5000 pg/ml.

Within this cohort, only 10.3% of patients underwent cardiology outpatient review within 28 days. This proportion increased with NT‐proBNP values: 5.7% (<400 pg/ml), 14.3% (400–2000 pg/ml), and 25.0% (for both 2000–5000 pg/ml and > 5000 pg/ml categories). The aforementioned time‐dependent Cox model (*Figure* *1*) also demonstrated that early cardiology review within 28 days was associated with significantly lower 1‐year mortality (aHR 0.47, 95% CI 0.39–0.57, *p* < 0.001). Propensity score matching (HR 0.73, 95% CI 0.64–0.83, *p* < 0.001) and landmark analysis (HR 0.51, 95% CI 0.41–0.64, *p* < 0.001) also showed a protective effect from early cardiology review. Of those who underwent cardiology review within 28 days, 75% had at least one further cardiology review in the next 12 months. On time‐dependent Cox modelling, multiple appointments were not significantly associated with a reduction in 1‐year mortality (aHR 0.896, *p* = 0.493).

Our findings confirm that NT‐proBNP is a powerful predictor of mortality in a broad clinical population, regardless of test indication, and that early cardiology involvement improves mortality. Despite this, only around one in four of these high‐risk patients (NT‐proBNP >2000 pg/ml) received early specialist outpatient review.

The mortality benefit of early cardiology review persisted across multiple analytical strategies designed to mitigate immortal time bias. These methods ensure accurate alignment of exposure with follow‐up: time‐dependent Cox models treat review as a time‐varying exposure, propensity score matching balances baseline covariates, and landmark analysis restricts comparisons to those alive at a fixed time point. Together, these suggest that early specialist input likely confers a genuine protective effect.

Whilst the beneficial effect of early cardiology involvement is most pronounced in those with significantly elevated NT‐proBNP levels (>2000 pg/ml), it should be noted a significant difference was evident in those with a ‘normal’ NT‐proBNP (<400 pg/ml). The reasons for referral in these cases are unclear, as test indications and clinical context were unavailable.

These findings have important implications for healthcare delivery models. Current approaches typically rely on symptomatic presentation and traditional referral pathways, which may delay specialist input until disease is advanced. Our data suggest that a more proactive approach, using NT‐proBNP as a tool to identify high‐risk patients for early intervention, could potentially improve outcomes. This aligns with emerging concepts of population health management and risk‐stratified care delivery.

Limitations of this study include the retrospective design, potential selection bias in testing and referral, and unmeasured confounding. Whilst the protective effect of early cardiology involvement remained when adjusting for age and sex, clinicians may have selected patients for referral based on factors such as comorbidities. Conversely, patients with more severe presentations may have been preferentially referred, potentially underestimating the benefit of cardiology review. Furthermore, data were not available to determine whether patients who did not receive early cardiology review were assessed in a timely manner in primary care, which may have led to underestimation of the observed benefit associated with early specialist input.

The indications for NT‐proBNP testing were not standardized and likely varied across clinical settings, including both acute and chronic presentations. Our study is limited by the lack of detailed clinical information regarding HF aetiology, comorbidities, and the specific interventions performed during cardiology consultations. Consequently, we cannot definitively determine which components of the specialist review contributed most to the improved outcomes. Although mortality data may not be entirely complete, the relatively closed nature of the island population means it is likely to be near‐comprehensive.

Despite these limitations, the large sample size and consistent findings across complementary methods reinforce the observed benefit of early cardiology involvement. Even accounting for residual confounding, the magnitude of benefit suggests that early cardiology involvement likely offers meaningful clinical advantage in high‐risk patients. These findings support the development of systematic approaches to identify and proactively manage patients with elevated NT‐proBNP, rather than relying solely on traditional referral pathways.

## Funding

Jersey Research Foundation and Jersey Community Foundation.

**Conflict of interest**: none declared.

## References

[1] Savarese G , Becher PM , Lund LH , Seferovic P , Rosano GMC , Coats AJS . Global burden of heart failure: A comprehensive and updated review of epidemiology. Cardiovasc Res 2023;118:3272–3287. 10.1093/cvr/cvac013 35150240

[2] Jones NR , Roalfe AK , Adoki I , Hobbs FDR , Taylor CJ . Survival of patients with chronic heart failure in the community: A systematic review and meta‐analysis. Eur J Heart Fail 2019;21:1306–1325. 10.1002/ejhf.1594 31523902 PMC6919428

[3] Echouffo‐Tcheugui JB , Zhang S , Daya N , McEvoy JW , Tang O , Juraschek SP , *et al*. NT‐proBNP and all‐cause and cardiovascular mortality in US adults: A prospective cohort study. J Am Heart Assoc 2023;12:e029110. 10.1161/JAHA.122.029110 37232235 PMC10382006

[4] Heidenreich PA , Bozkurt B , Aguilar D , Allen LA , Byun JJ , Colvin MM , *et al*. 2022 AHA/ACC/HFSA guideline for the management of heart failure: A report of the American College of Cardiology/American Heart Association Joint Committee on Clinical Practice Guidelines. Circulation 2022;145:e895–e1032. 10.1161/CIR.0000000000001063 35363499

[5] Public Health Intelligence . Public Health Reports: Government of Jersey. 2025 (cited 1 Sept 2025). https://www.gov.je/Health/PublicHealth/Pages/StatesReports.aspx

